# Single-Atomic Site Catalyst Enhanced Lateral Flow Immunoassay for Point-of-Care Detection of Herbicide

**DOI:** 10.34133/2022/9823290

**Published:** 2022-08-21

**Authors:** Zhaoyuan Lyu, Shichao Ding, Peter Tieu, Lingzhe Fang, Xin Li, Tao Li, Xiaoqing Pan, Mark H. Engelhard, Xiaofan Ruan, Dan Du, Suiqiong Li, Yuehe Lin

**Affiliations:** ^1^School of Mechanical and Material Engineering, Washington State University, Pullman, WA 99164, USA; ^2^Department of Chemistry, University of California, Irvine, Irvine, CA 92697, USA; ^3^Department of Chemistry and Biochemistry, Northern Illinois University, DeKalb, IL 60115, USA; ^4^X-ray Science Division, Argonne National Laboratory, Lemont, IL 60439, USA; ^5^Irvine Materials Research Institute (IMRI), Department of Materials Science and Engineering, University of California, Irvine, Irvine, CA 92697, USA; ^6^Environmental Molecular Sciences Laboratory, Pacific Northwest National Laboratory, Richland, WA 99354, USA; ^7^DL ADV-Tech, Pullman, WA 99163, USA

## Abstract

Point-of-care (POC) detection of herbicides is of great importance due to their impact on the environment and potential risks to human health. Here, we design a single-atomic site catalyst (SASC) with excellent peroxidase-like (POD-like) catalytic activity, which enhances the detection performance of corresponding lateral flow immunoassay (LFIA). The iron single-atomic site catalyst (Fe-SASC) is synthesized from hemin-doped ZIF-8, creating active sites that mimic the Fe active center coordination environment of natural enzyme and their functions. Due to its atomically dispersed iron active sites that result in maximum utilization of active metal atoms, the Fe-SASC exhibits superior POD-like activity, which has great potential to replace its natural counterparts. Also, the catalytic mechanism of Fe-SASC is systematically investigated. Utilizing its outstanding catalytic activity, the Fe-SASC is used as label to construct LFIA (Fe-SASC-LFIA) for herbicide detection. The 2,4-dichlorophenoxyacetic acid (2,4-D) is selected as a target here, since it is a commonly used herbicide as well as a biomarker for herbicide exposure evaluation. A linear detection range of 1-250 ng/mL with a low limit of detection (LOD) of 0.82 ng/mL has been achieved. Meanwhile, excellent specificity and selectivity towards 2,4-D have been obtained. The outstanding detection performance of the Fe-SASC-LFIA has also been demonstrated in the detection of human urine samples, indicating the practicability of this POC detection platform for analyzing the 2,4-D exposure level of a person. We believe this proposed Fe-SASC-LFIA has potential as a portable, rapid, and high-sensitive POC detection strategy for pesticide exposure evaluation.

## 1. Introduction

The broad usage and high consumption of herbicide have resulted in the accumulation of herbicide in the soil, air, ocean, food, water sources, and even in the human body, severely impacting the ecosystem and food safety [1]. Nowadays, researchers have found that exposure to herbicide can be associated with serious health problems such as endocrine-disrupting activities, [2] acute congestion, and degenerative changes in the central nerve system [3]. 2,4-Dichlorophenoxyacetic acid (2,4-D), a common and important phenoxy herbicide, was the first successful one ever developed since its commercial release in the 1940s [4]. It is considered as one of the “probably carcinogenic compounds to humans” [5, 6]. Since 2,4-D remains chemically stable in the human body and can be secreted through urine [7], direct detection of 2,4-D concentration in human urine is a meaningful and effective way to judge a person's exposure level. For example, for nonexposed adults and children in the USA and Canada, 2,4-D concentrations in urine samples are less than 3.5 ng/mL [8, 9], while for the workers who are occupationally exposed to herbicides, the average 2,4-D concentrations of 7.8 and 33 ng/mL were detected in urine before and after 2,4-D spray, respectively [10, 11]. Up to now, the quantification of 2,4-D and other herbicides is usually based on its inhibitory effect on alkaline phosphatase (ALP) activity via electrochemical workstations or various chromatographic techniques [12, 13]. However, these methods suffer from poor reproducibility, expensive equipment, meticulous sample preparation, and highly qualified technicians, which limit their fast point-of-care (POC) applications [13]. Hence, developing effective methods for simple, rapid, cost-effective, and sensitive detection of 2,4-D becomes an important goal.

Lateral flow immunoassay (LFIA), as an effective POC detection method, is widely reported for monitoring and diagnosis of various specific targets [14]. However, the drawback of low sensitivity limits its further practical applications. In the past few decades, the continuous development of nanotechnology has brought vitality to the development of various nanosized materials with enzyme-like activities [15, 16]. Having been introduced as the label for signal amplification since 2015 [17], these kinds of nanomaterials have allowed LFIA to achieve high-sensitive detection and thus greatly benefited to trace detections. Our group has reported that the enzyme-like nanomaterial-linked LFIA could boost signal amplification for biosensing. An obvious color change on concerned lines can be produced through catalyzing substrates by the intrinsic peroxidase-like activity of nanomaterials; thus, the detection performance is greatly improved [18, 19]. Though the stability of enzyme-like nanomaterials is much better than their natural counterparts, substantially improving the catalytic activity of this type of nanomaterial still remains a grand challenge.

Regulating the size of nanomaterials, especially downsizing the catalytically active sites on materials to several or even single atoms, has been considered as a potential way to address this challenge. Single-atom site catalysts (SASCs) have recently attracted widespread attention due to their isolated active metal sites and unique electronic/geometric structures [20–23]. Different from traditional nanomaterials where the catalytic activity mainly comes from surface atoms, the increased catalytic abilities of SASCs come from almost one hundred percent utilization of active metal atoms [24]. Most importantly, the coordination structure of Fe-N_x_ sites on SASCs vividly mimics the active sites of natural enzyme [25–28]. Based on these, SASCs not only show a myriad of advantages in electrocatalysis applications [29–32] but also exhibit high-performance enzyme-like properties and huge potential in biosensing applications [33–35]. Ouyang et al. prepared the Co single-atom catalysts (SACs) through facile solvothermal method without high-temperature calcination of Co/Fe bimetallic metal-organic framework. And Co-SASCs was used as signaling probes for chemiluminescence immunoassay of cardiac troponin I; the detection limit of the target analyte is 3.3 pg mL^−1^ [36]. Our previous work developed Fe-N_x_ SASC to replace natural enzymes in a commercial enzyme-linked immunosorbent assay (ELISA) for early-stage detection of Alzheimer's disease. The designed SASC-labeled linked immunosorbent assay is over ten times more sensitive than that of commercial ELISA [37]. Therefore, we believe that the SASCs with enhanced catalytic activity and stability can be an ideal substitute for traditional nanomaterials in LFIA to improve their detection ability.

**Figure 1:**
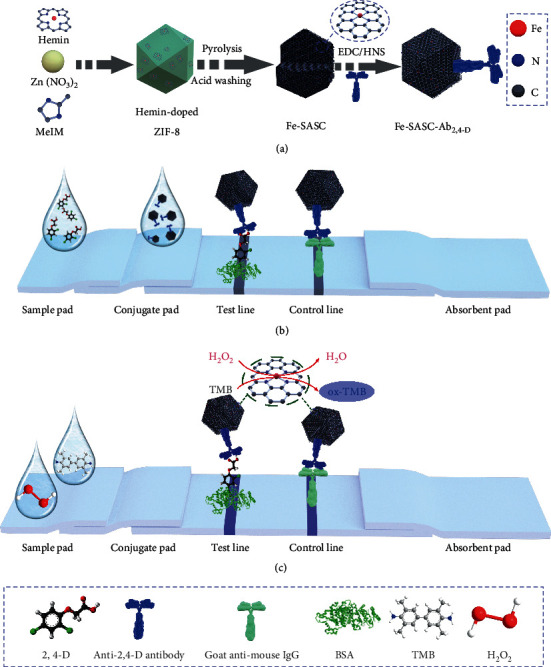
Schematic illustration of (a) preparation process of the Fe-SASC and Fe-SASC-Ab_2,4-D_; (b) Fe-SASC enhanced competitive lateral flow immunoassay (Fe-SASC-LFIA) for detection of 2,4-D.

In this work, we synthesize a single-atom site iron catalyst (Fe-SASC) and use it in competitive LFIA for POC detection of herbicide. Herein, hemin is selected as Fe precursor owing to the existing similar Fe coordination site of the natural enzyme. Owing to the nanoconfinement effect of ZIF-8, the doped hemin sites could be directly converted to peroxidase-like active center, and its atomically dispersed iron active sites were proved by aberration-corrected scanning transmission electron microscopy (AC-STEM) image and extended X-ray absorption fine structure (EXAFS). A series of characterization were conducted to reveal the POD-like activity and catalytic mechanism of Fe-SASC. The 2,4-dichlorophenoxyacetic acid (2,4-D) is selected as a kind of herbicide target in this work. The designed Fe-SASC labeled LFIA (Fe-SASC-LFIA) detection platform is illustrated in Figure 1. When 2,4-D is not present in the sample, Fe-SASC labeled 2,4-D antibody (Fe-SASC-Ab_2,4-D_) will be captured by the test lines where corresponding haptens (BSA-2,4-D) are immobilized, which the visible label will be displayed (negative). Conversely, when 2,4-D exists in the sample, it will bind to the antibodies to prevent them from binding to the haptens immobilized on the test line so that no visible label will appear. The Fe-SASC has superior catalytic activity and can be used as an antibody label to achieve the biorecognition process and amplify the signal. Highly sensitive and specific detection of 2,4-D in human urine was demonstrated by using the developed Fe-SASC-LFIA. The results prove that the Fe-SASC-LFIA exhibits excellent sensitivity and practical applicability, making the fabricated bioassays with satisfactory detection ability and feasibility in clinical diagnosis.

## 2. Results and Discussion

### 2.1. Synthesis and Structure Characterization of Fe-SASC

**Figure 2:**
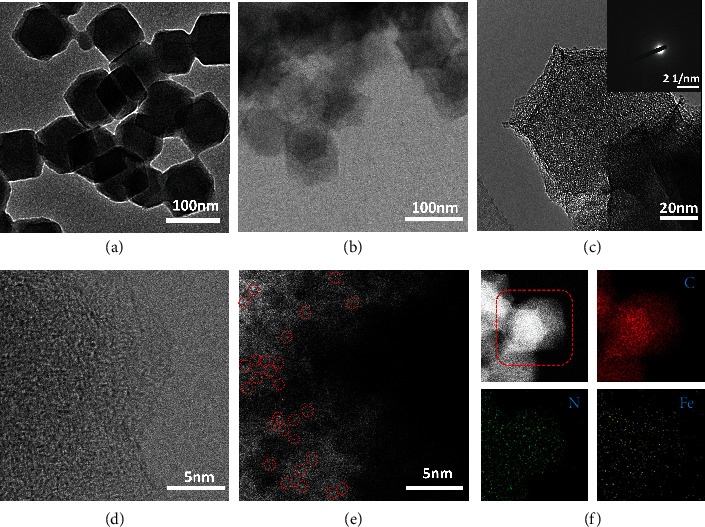
(a) TEM image of hemin-doped ZIF-8 precursor. (b, c) Low- and high-magnification TEM images of Fe-SASC. Inset in (c): the SAED pattern of Fe-SASC. (d, e) BF-STEM and HAADF-STEM image of Fe-SASC. Red circles indicate single atoms of Fe. (f) HAADF-STEM image and the corresponding EDS elemental mapping of Fe-SASC.

The preparation of hemin-doped ZIF-8 precursor is similar to the previous report except for adding a certain amount of hemin, as shown in Figure 2(a) [38]. The synthesis details are presented in Experimental. Figures 2(a)–2(c) exhibit the morphologies of the hemin-doped ZIF-8 precursor and the synthesized Fe-SASC, respectively. Similar to the hemin-doped ZIF-8 precursor, the dodecahedral structure is well maintained after the pyrolysis process to form the Fe-SASC. The average size of the synthesized Fe-SASC is around 100 nm, and such a small size meets the liquidity requirements of the LFIA sensing process. The selected area electron diffraction (SAED) pattern in Figure 2(c) shows no diffraction spots, which means no crystalline phase is present in Fe-SASC. The nanocrystal-free feature is also evidenced by X-ray diffraction (XRD) pattern (Figure S1). The bright-field scanning TEM (BF-STEM) image in Figure 2(d) confirms that Fe-SASC is composed of distorted graphitic carbon without any metal clusters. These analysis results support that catalytically active sites of SASC are formed at the single Fe atomic level. Such active sites are believed rich in high specific surface area and vast nanopores of the SASC. Besides, due to the doping of macromolecule hemin as the Fe precursor, lots of micropores are also formed here to improve the specific surface area and enhance catalytic efficiency [39]. Also, a related low ratio of C-sp^2^ in C 1s spectrum in Figure S2 demonstrates lots of distorted graphitic carbon species existed in Fe-SASC [40]. The specific surface area of Fe-SASC was evaluated by N_2_ adsorption-desorption measurement, which is calculated around 664.91 m^2^ g^−1^ from Brunauer-Emmett-Teller (BET) method. The corresponding isotherm curve (Figure S3) indicates that Fe-SASC has both micropores and mesopores, supported by the obvious adsorption and hysteresis in the low-pressure zone (*P*/*P*_0_ = 0-0.1). Nonlocal density functional theory (NLDFT) was also carried out to analyze the specific pore distributions (Figure S4). Application of high-angle annular dark-field STEM (HAADF-STEM) imaging was applied to study the atomic level structure of Fe-SASC (Figure 2(e)). The observed isolated bright spots are circled by red circles, indicating that plentiful single atom Fe sites are anchored on Fe-SASC. In addition, the energy-dispersive X-ray spectroscopy (EDS) elemental mapping is applied to verify that the C, N, and Fe elements are uniformly distributed on the nanostructure (Figure 2(f)).

**Figure 3:**
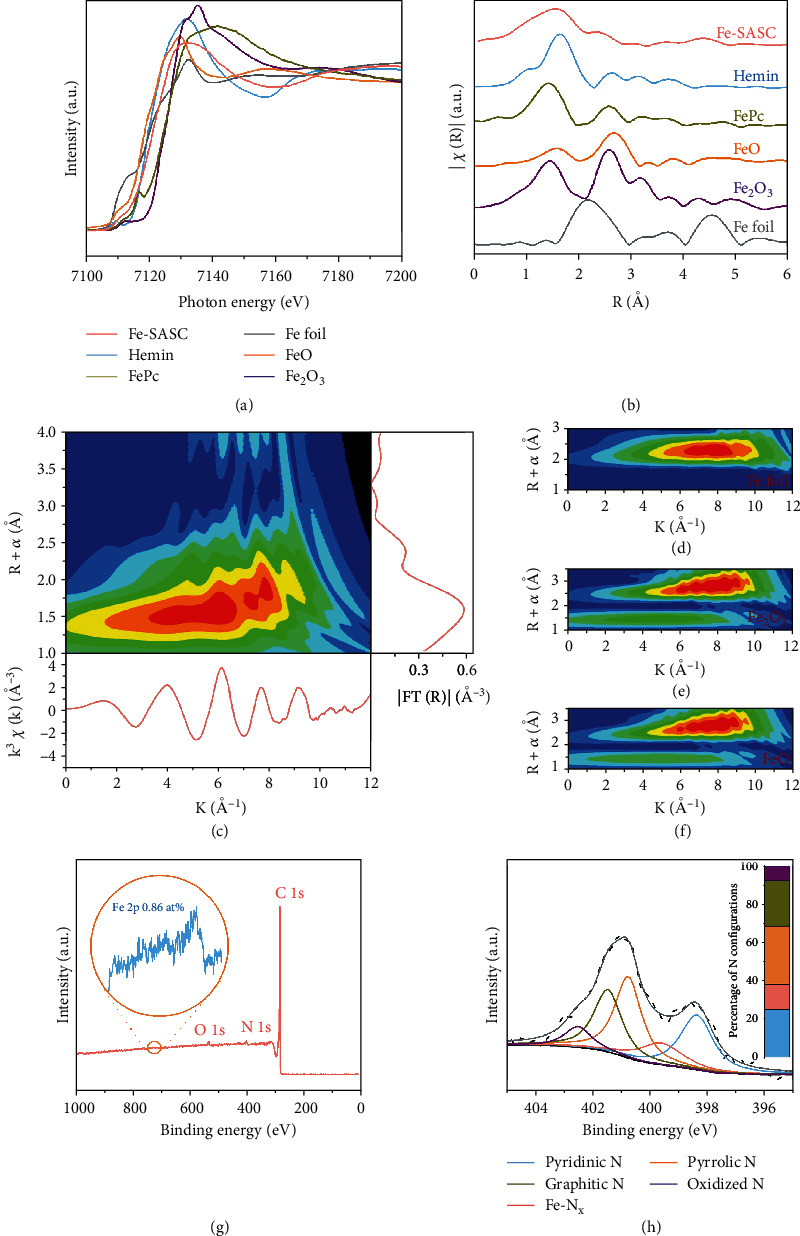
(a) Fe K-edge X-ray absorption near-edge structure (XANES) spectrum of Fe-SASC and reference samples of hemin, FePc, Fe foil, FeO, and Fe_2_O_3_. (b) FT k^3^-weighted extended X-ray absorption fine structure (EXAFS) spectrum of Fe-SASC, hemin, FePc, Fe foil, FeO, and Fe_2_O_3_. (c) Full-range WT representation of the EXAFS signal for a representative Fe-SASC sample. (d–f) WT of Fe foil, Fe_2_O_3_, and FeO, respectively. (g) XPS survey spectra with an inset of the high-resolution Fe 2p spectrum. (h) N 1s spectrum of Fe-SASC. Inset: the percentage of N 1s configuration.

The chemical state and coordination environment of Fe center were investigated by X-ray absorption spectroscopy analysis, and the X-ray absorption near-edge structure (XANES) spectra reveal that the absorption edge position of Fe-SASC is between FeO and Fe_2_O_3_, indicating that the average valence state of Fe atoms is between Fe^+2^ and Fe^+3^ (Figure 3(a)). In Figure 3(b), Fourier transforms (FT) from extended X-ray absorption fine structure (EXAFS) indicate that Fe-SASC only exhibits a prominent peak at 1.57 Å, which corresponds to the Fe-N first coordination shell in the hemin reference sample. Most importantly, no obvious Fe-Fe peak (2.22 Å) nor other high-shell peaks are observed, confirming that Fe in Fe-SASC exists as the isolated atom form [41]. It is worth noting that the k-space EXAFS oscillation spectrum of Fe-SASC (Figure S5) is different from that of Fe foil and Fe oxides, but similar to Fe single-atom references of hemin and iron (II) phthalocyanine (FePc). Moreover, Fe K edge EXAFS oscillations were analyzed by Wavelet transform (WT). In Figure 3(c), from the WT contour plots, only one intensity maximum at about 5.5 Å^−1^ can be observed, and no Fe-Fe intensity maximum corresponding is detected compared with the WT plots of Fe foil, Fe_2_O_3_, and FeO (Figures 3(d)–3(f)). X-ray photoelectron spectroscopy (XPS) analysis was conducted to evaluate the chemical composition of iron atoms, and results show that the quantified Fe content in Fe-SASC is about 0.86 at% (Figure 3(g)). Also, inductively coupled plasma mass spectrometry (ICP-MS) was further used to detect the Fe content and 2.45 wt % Fe was detected in Fe-SASC. The high-resolution N 1s is shown in Figure 3(h); the peaks near 398.4, 399.6, 401.5, and 402.5 eV can be divided into pyridinic, pyrrolic, graphitic, and oxidized N, respectively, which confirms that the nitrogen is indeed incorporated into the carbon matrix [42]. Based on binding energy, a 400.8 eV spectral valley between two main peaks of pyridinic and pyrrolic can be attributed to Fe-N_x_ species [26, 43]. The inset of Figure 3(h) indicates that the percentage of Fe-N_x_ configuration is 13.2% in all doped N species.

### 2.2. Peroxidase-Like Properties of Fe-SASC

**Figure 4:**
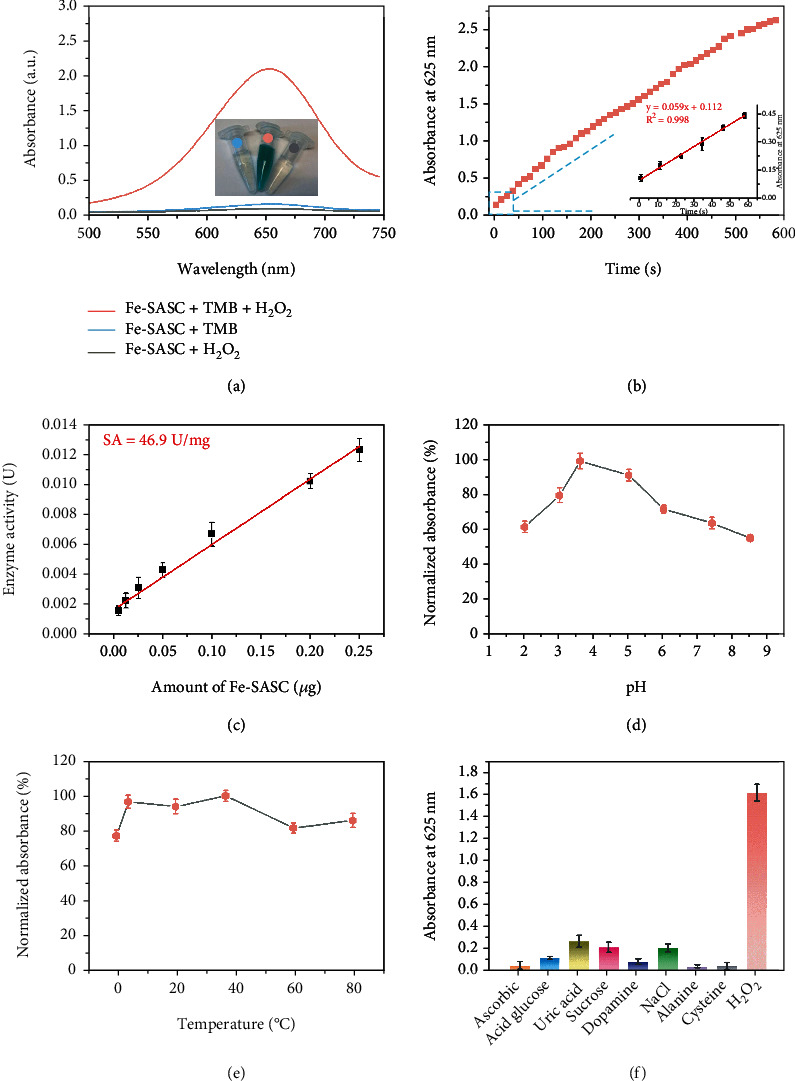
(a) Absorption curves of the Fe-SASC in the solution of TMB, H_2_O_2_, and TMB+H_2_O_2_, respectively. Inset: photographs of the color changes. (b) TMB chromogenic reaction curve of absorbance to time catalyzed by Fe-SASC and the inset is the initial linear portion. (c) The relationship between specific activity (SA) of Fe-SASC and its amount. The peroxidase-like catalytic activity of Fe-SASC against various (d) pH and (e) temperature values. (f) Specificity evaluation of Fe-SASC for various interferences.

Peroxidase-like properties of Fe-SASC were verified by typical chromogenic reactions, in which 3,3′,5,5′-tetramethylbenzidine (TMB) was used as substrates. As shown in Figure 4(a), the obvious color change and enhanced absorbance intensity prove that TMB is oxidized by the Fe-SASC in the presence of H_2_O_2_. The typical absorbance curve of TMB-Fe-SASC chromogenic reaction within 600 s is presented in Figure 4(b), where the absorbance increases with reaction time. In the first 60 s, linear regression analysis can confirm a linear reaction between absorbance and time (*R*^2^ = 0.998). These results indicate that the Fe-SASC possesses excellent POD-like characteristics and can be used in bioapplications. Catalytic activity expressed in units (U) of Fe-SASC was calculated (Figure 4(c)). The specific activity (SA) was determined to be 46.9 U mg^−1^, which is higher than most conventional nanomaterials (Table S1), further proving the unprecedented POD-like activities of the synthesized Fe-SASC. This high activity originated from the structural similarity between Fe-SASC and effective structure in natural enzymes [26, 37]. To evaluate the potential effects of various harsh environments on Fe-SASC, the catalytic activity of Fe-SASC was measured under different pH values and temperatures (Figures 4(d) and 4(e)). The Fe-SASC can maintain its high activity in a wide range of pH values and temperatures. Fe-SASC has the maximum activity under the pH value of 3.5 as well as keeps above 50% activity under different pH values. The Fe-SASC can also preserve over 70% of its activity from 4 to 80°C and exhibit the highest catalytic activity at 37°C. These results demonstrate that the Fe-SASC can maintain stability under harsh environments. Finally, the steady-state kinetic curves of Fe-SASC towards TMB substrates and H_2_O_2_ were obtained by fitting with the Michaelis-Menten equation, as shown in Figure S6, and the corresponding Michaelis-Menten parameters are listed in Table S2. Compared to natural horseradish peroxidase (HRP) [34], Fe-SASC showed comparable *K*_*m*_ towards H_2_O_2_ and relatively low *K*_*m*_ towards TMB, indicating that Fe-SASC has a superior binding affinity toward TMB and a similar affinity level towards H_2_O_2_. Lastly, the selectivity of Fe-SASC towards H_2_O_2_ was analyzed. As shown in Figure 4(f), the presence of H_2_O_2_ results in a distinct signal, while other competing interference can only produce negligible signals, indicating the satisfactory selectivity of Fe-SASC.

### 2.3. Mechanisms for Peroxidase-Like Activity of Fe-SASC

**Figure 5:**
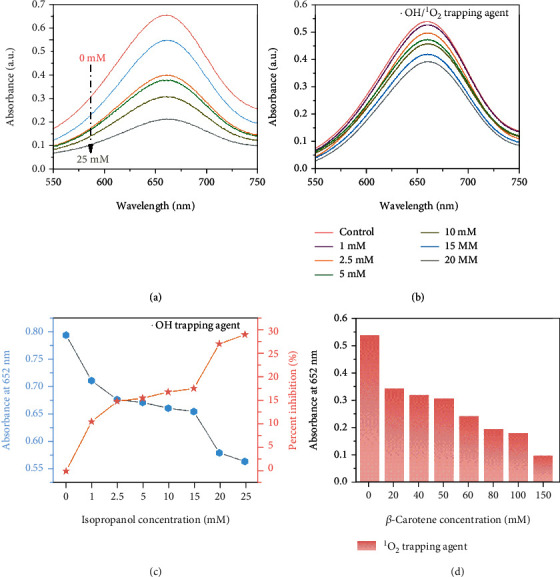
Absorption curves upon the addition of various concentrations of (a) KSCN and (b) NaN_3_ as the ·OH/^1^O_2_ scavenger. (c) Absorption values and their corresponding inhibition percentage after adding isopropanol. (d) Absorption after adding different amounts of *β*-carotene.

It is speculated that the excellent POD-like activity of Fe-SASC originates from the reactive oxygen species (ROS) generated by the decomposition of H_2_O_2_ on the complex. Based on this, we conducted a series of chemical experiments to study the mechanism of the catalytic process of POD-like Fe-SASC. Firstly, the role of single atom Fe in catalytic efficiency was verified by thiocyanate ions (SCN^−^). A stable chelate complex can be formed between SCN^−^ and Fe-centered catalytic sites, thus blocking Fe activity sites and preventing decomposition of H_2_O_2_. As shown in Figure 5(a), absorbance spectrums drop dramatically as more SCN^−^ is added, indicating that the atomically dispersed Fe-N_x_ active sites are the main source of the POD-like activity of Fe-SASC. Then, various scavengers were used to study the active intermediates involved in the POD-like process (Figures 5(b)–5(d)). The intermediate ·OH/^1^O_2_ was verified in Figure 5(b). The absorbance value of ox-TMB decreased with increasing amounts of the added NaN_3_, showing that ·OH/^1^O_2_ is involved in the oxidation coloration reaction [44]. Isopropanol and *β*-carotene were further used to confirm the existence of ·OH and ^1^O_2_, respectively (Figures 5(c) and 5(d)). According to the above results, the oxidation coloration reaction is the result of the combined effect of ·OH and ^1^O_2_. The strong oxidizing properties of ROS can effectively oxidize the chromogenic substrate.

### 2.4. Optimization for the Fe-SASC Labeled Ab_2,4-D_ for LFIA Application

**Figure 6:**
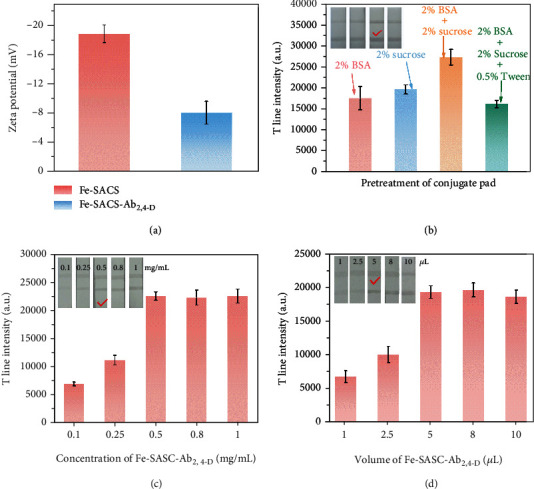
(a) Zeta potential analysis of Fe-SASC and Fe-SASC-Ab_2,4-D_ conjugates. (b) Optimization of pretreatment of the conjugate pads with various solutions. (c, d) Optimization of loading amount of concentration and volume of Fe-SASC-Ab_2,4-D_ applied on the sample pad, respectively.

The prepared Fe-SASCs were conjugated with Ab_2,4-D_ and used as the signal labels for 2,4-D detection in competitive LFIA. The properties of the labeled antibody (Fe-SASC-Ab_2,4-D_) were investigated by zeta potential characterization (Figure 6(a)). The reduced negative charge of Fe-SASC demonstrating the Ab_2,4-D_ is successfully labeled with Fe-SASC. In LFIA application, the pretreatment of conjugate pads and the loading amount of Fe-SASC-Ab_2,4-D_ probes are crucial factors in obtaining the best detection performance. For example, pretreatment of the conjugate pad will affect the flow rate of the probes to test line (T-line) and control line (C-line) on NC membrane, ultimately affecting the processing time and sensitivity. Herein, the LFIA assembling process and Fe-SASC-Ab_2,4-D_ loading parameter were optimized. The sample pad treated with 2% BSA+2% sucrose was chosen since it has the highest T-line intensity, indicating the lower nonspecific adsorption on the conjugate pad and satisfactory delivery of probes to the T-line area is achieved (Figure 6(b)). Moreover, the loading amount of Fe-SASC-Ab_2,4-D_ probes also plays an important role because the visualization of the test area is due to the accumulation of probes on the T-line and C-line. Excessive loading of Fe-SASC-Ab_2,4-D_ probes will increase background noise and limit the detection sensitivity, while an insufficient amount will cause weak signal which cannot be discerned compared to the C-line or cannot be visualized [45]. As seen in Figure 6(c), the T-line intensity increased along with the increasing concentration or volume of Fe-SASC-Ab_2,4-D_ at first. No obvious difference in T-line intensity is observed when the concentration is above 0.5 mg/mL. After setting the Fe-SASC-Ab_2,4-D_ concentration at 0.5 mg/mL, the volume was also investigated and determined as 5 *μ*L, as shown in Figure 6(d). Therefore, based on these optimized results, 5 *μ*L of 0.5 mg/mL probe is selected as the suitable loading parameters for this system.

### 2.5. Detection Performances of the Fe-SASC-Based LFIA

**Figure 7:**
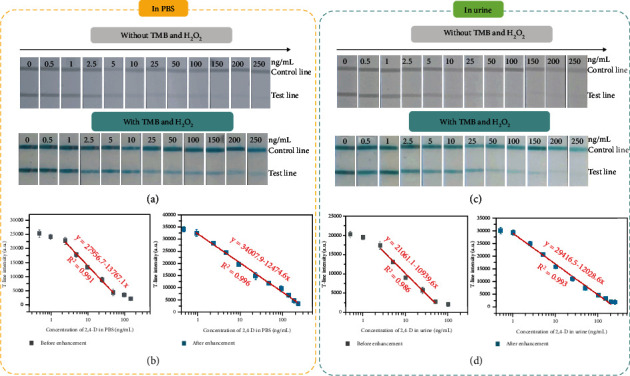
Detection performance of Fe-SASC-LFIA for 2,4-D detection. (a, c) Typical photographs of Fe-SASC-LFIA in the presence of different 2,4-D levels in PBS and in human urine before and after Fe-SASC enhancement, respectively. (b, d) Relationship between the T-line intensity and various concentrations of 2,4-D in PBS and in human urine, respectively.

The optimized Fe-SASC-LFIA was used to detect 2,4-D in standard solutions (in PBS) and human urine samples, and the detection performance of the LFIA was systematically evaluated.  Figure 7(a) shows the photographs of test/control lines before and after the Fe-SASC enhancement for 2,4-D detection with different 2,4-D concentrations ranging from 0 ng/mL to 250 ng/mL in PBS. For negative samples (no 2,4-D is presented), there is no color difference between T-line and control line that can be observed. For positive samples, the color on T-line is inversely proportional to the 2,4-D concentration and T-line becomes invisible when the 2,4-D concentration is high enough, which is consistent with competitive immunoreactions. The dramatic enhancement of the detection signal is achieved through the reaction between the TMB and Fe-SASC. After adding TMB/H_2_O_2_, the T-line and control line colors change from light gray (color from Fe-SASC) to bright blue (results from Fe-SASC's catalyzing effect), which significantly enhances the signal output and dramatically widen the detection range. The accurate quantitative analysis of 2.4-D detection by Fe-SASC-LFIA is conducted by a digital camera and processed with ImageJ software (software processing steps are illustrated in Figure S7). As shown in Figure 7(b), there is a linear correlation between the gray T-line intensity and the concentration of 2,4-D in the range of 2.5 to 50 ng/mL before adding TMB/H_2_O_2_. The limit of detection (LOD) is defined as the concentration of 2,4-D corresponding to the signal intensity, which is calculated by ^−^*S* − 3*σ* (^−^*S* is the average signal intensity of 0 ng/mL measured 10 times, and *σ* is the standard deviation) [46]. LOD of Fe-SASC-LFIA is determined to be 1.54 ng/mL. After the signal enhancement via adding TMB/H_2_O_2_, we can see a much wider detection ranging from 1 to 250 ng/mL with a lower LOD of 0.82 ng/mL in the Fe-SASC-LIFA. The improved sensitivity and LOD can be attributed to the enhanced signal generated by the POD-like activity of Fe-SASC. Such excellent detection performance of Fe-SASC-LFIA for 2,4-D detection is also better than other published works (Table S3). We also analyzed the detection ability in real human urine samples, as shown in Figures 7(c) and 7(d). Good linear relations in the range of 2.5 to 50 ng/mL with LOD of 1.82 ng/mL and 1 to 200 ng/mL with LOD of 0.93 ng/mL before and after Fe-SASC enhancement are obtained, respectively. Comparable detection performance was achieved for detection of PBS-based standard solutions and detection of urine samples, proving that the urine matrix has a limited effect on the detection performance of Fe-SASC-LFIA. Moreover, the selectivity of this assay for 2,4-D in human urine was verified by evaluating the impact of interfering substances such as Na^+^, K^+^, Cl^−^, glucose, and uric acid (UA) (Figure S8) [47]. Obvious color change on T-line is observed when in the presence of 2,4-D, while other interfering substances are ineffective, indicating an outstanding selectivity of the proposed Fe-SASC-LFIA towards 2,4-D against potential interfering substances. Spike-recovery experiments evaluated the analytical reliability and accuracy of Fe-SASC-LFIA. Different concentrations of 2,4-D spiked human urine samples were prepared with satisfactory recoveries of 2,4-D in the range from 94.4 to 113.4% are obtained (Table S4). The high recovery level and low variability results indicate the reliability and accuracy of proposed Fe-SASC-LFIA on 2,4-D detection in actual samples.

## 3. Conclusion

In summary, a single-atom iron catalyst (Fe-SASC) has been developed for rapid and ultrasensitive POC detection of a herbicide (2,4-D). As expected, Fe-SASC has excellent peroxidase-like activity and exceptional stability due to the maximum utilization of active metal atoms and the structural mimicry of the active sites of the natural enzyme. The Fe-SASC with high POD-like activity can trigger the colorimetric reaction of the chromogenic substrate to enhance the signal intensity, thus significantly improving the detection performance of LFIA. High-performance detection of 2,4-D with a low detection limit (0.82 ng/mL), wide detection range (1-250 ng/mL), and good selectivity have been demonstrated. Furthermore, the proposed Fe-SASC-LFIA exhibits high specificity and satisfactory recovery. The excellent detection abilities are sustained in the real human urine samples, further demonstrating its potential POC practicability.

## Data Availability

All data needed to evaluate the conclusions in the paper are present in the paper and/or the Supplementary Materials. Additional data are available from the corresponding author upon reasonable request.

## Supplementary Materials

Supplementary Materials Additional experiment details. Figure S1: XRD pattern of Fe-SASC. Figure S2: high-resolution C 1s spectra of Fe-SASC. Figure S3: the N_2_ adsorption/desorption isotherm curves of Fe-SASC. Figure S4: pore distribution of Fe-SASC by NLDFT. Figure S5: EXAFS oscillations of Fe-SASC and reference samples of hemin, FePc, Fe foil, FeO, and Fe_2_O_3_ at k-space. Figure S6: steady-state kinetic curves of Fe-SASC toward (a) TMB and (b) H_2_O_2_. Figure S7: image analysis procedure by ImageJ software. (a) Test strips captured by a camera after 10 min of lateral flow assay, (b) picture converted to an 8-bit grayscale image in ImageJ software, (c) the Gel Analyzer function of the chosen rectangular regions surrounding the test lines, (d) the signal strength peak generated by the Gel Analyzer function in ImageJ. Figure S8: (a, b) photographs of Fe-SASC-LFIA towards 2,4-D and interference substances in human urine before and after Fe-SASC enhancement, respectively. (c) Corresponding T-line signal intensities (concentrations of interfering substances were all 100 ng/mL, 2,4-D was 25 ng/mL). Table S1: comparison of peroxidase-mimic activity of Fe-SASC and reported nanomaterials and HRP. Table S2: comparison of steady-state kinetic parameters of Fe-SASC and natural HRP. Table S3: comparison of our work and other different methods for 2,4-D detection. Table S4: spiked-recovery test of plasma samples using the proposed Fe-SASC-LFIA in human urine (*n* = 3).
