# Supplementary material for: Single-Atomic Site Catalyst Enhanced Lateral Flow Immunoassay for Point-of-Care Detection of Herbicide
Source: Research (Wash D C). 2022 Aug 21;2022:9823290. doi: 10.34133/2022/9823290 (PMC9435159; doi:10.34133/2022/9823290)
Supplement: Supplementary Materials — Additional experiment details. Figure S1: XRD pattern of Fe-SASC. Figure S2: high-resolution C 1s spectra of Fe-SASC. Figure S3: the N2 adsorption/desorption isotherm curves of Fe-SASC. Figure S4: pore distribution of Fe-SASC by NLDFT. Figure S5: EXAFS oscillations of Fe-SASC and reference samples of hemin, FePc, Fe foil, FeO, and Fe2O3 at k-space. Figure S6: steady-state kinetic curves of Fe-SASC toward (a) TMB and (b) H2O2. Figure S7: image analysis procedure by ImageJ software. (a) Test strips captured by a camera after 10 min of lateral flow assay, (b) picture converted to an 8-bit grayscale image in ImageJ software, (c) the Gel Analyzer function of the chosen rectangular regions surrounding the test lines, (d) the signal strength peak generated by the Gel Analyzer function in ImageJ. Figure S8: (a, b) photographs of Fe-SASC-LFIA towards 2,4-D and interference substances in human urine before and after Fe-SASC enhancement, respectively. (c) Corresponding T-line signal intensities (concentrations of interfering substances were all 100 ng/mL, 2,4-D was 25 ng/mL). Table S1: comparison of peroxidase-mimic activity of Fe-SASC and reported nanomaterials and HRP. Table S2: comparison of steady-state kinetic parameters of Fe-SASC and natural HRP. Table S3: comparison of our work and other different methods for 2,4-D detection. Table S4: spiked-recovery test of plasma samples using the proposed Fe-SASC-LFIA in human urine (n = 3). [file 9823290.f1.docx]

**Single-Atomic Site Catalyst Enhanced Lateral-flow Immunoassay for Detection of Herbicide**

Zhaoyuan Lyu,^a,1^ Shichao Ding,^a,1^ Peter Tieu,^c^ Lingzhe Fang,^d^ Xin Li,^a^ Tao Li,^d, e^ Xiaoqing Pan,^f^ Mark H. Engelhard,^g^ Xiaofan Ruan,^a^ Dan Du,^a,*^ Suiqiong Li,^b,*^ Yuehe Lin^a,*^

^a^ School of Mechanical and Material Engineering, Washington State University, Pullman, WA, 99164, USA

^b^ DL ADV-Tech, Pullman, WA, 99163, USA

^c^ Department of Chemistry, University of California, Irvine, Irvine, CA 92697, USA

^d^ Department of Chemistry and Biochemistry, Northern Illinois University, DeKalb, IL, 60115, USA

^e^ X-ray Science Division, Argonne National Laboratory, Lemont, IL, 60439, USA

^f^ Irvine Materials Research Institute (IMRI); Department of Materials Science and Engineering, University of California, Irvine, Irvine, CA, 92697, USA

^g^ Environmental Molecular Sciences Laboratory, Pacific Northwest National Laboratory, Richland, WA, 99354, USA

^1^ These authors contributed equally

*Corresponding Authors. E-mail: [yuehe.lin@wsu.edu](mailto:yuehe.lin@wsu.edu); [dladvtech@gmail.com](mailto:dladvtech@gmail.com); [annie.du@wsu.edu](mailto:annie.du@wsu.edu)

**Characterization**

The materials were characterized using transmission electron microscopy (TEM, Tecnai F20, 200 kV; Philips CM200 UT, 200 kV; JEOL ARM300CF, 300 kV), X-ray photoelectron spectroscopy (XPS, ThermoFisher Thermo, Al Kα), and X-ray diffraction (XRD, Rigaku Miniflex 600, 40 kV). Nitrogen gas adsorptions were done using a Micromeritics ASAP 2020Plus at 77 K. The X-ray absorption spectroscopy measurements at the Fe K-edge (7,112 eV) were performed at the Advanced Photon Source (APS) on the bending-magnet beamline 12-BM and 20-BM. A Fe metal foil was used to calibrate the X-ray beam energy and was simultaneously measured with each sample. The radiation was monochromatized by a Si (111) double-crystal monochromator. The harmonic rejection was accomplished with a Harmonic rejection mirror. The single-atomic site catalyst (SASC)sample spectra were collected in fluorescence mode by vortex four-element silicon drift detector, and the standards (including the foil) have been measured in transmission mode. XAS data reduction and analysis were processed by Athena software. For wavelet transform analysis, the χ(k) exported from Athena was input to the Hama Fortran code. The parameters were set as follows: k weight, 2; R range, 1-4 Å; k range, 0-10 Å^-1^.

**Experimental Section**

*Chemicals*

2-Methylimidazole (MeIM), hemin (from bovine, ≥90%), bovine serum albumin (BSA), hydrogen peroxide (H_2_O_2_), 3,3′,5,5′-tetramethylbenzidine (TMB), N-(3-dimethylamino propyl)-N′-ethylcarbodiimide hydrochloride (EDC), N-hydroxysuccinimide (NHS) and dimethyl sulfoxide acquired from Sigma-Aldrich, USA; Methyl Alcohol (anhydrous) was purchased from Avantor Performance Materials, USA; Zinc nitrate hexahydrate (Zn(NO_3_)_2_·6H_2_O, 99%) was provided by Alfa Aesar, USA; 2,4-Dichlorophenoxyacetic acid antigen (2,4-D BSA) was provided by Biorbyt Inc. USA, Anti-2,4-D antibody (Ab_2,4-D_) was obtained from Invitrogen, USA, and Goat anti-mouse IgG antibody was purchased from CosmoBio. CO., LTD., USA. Phosphate buffered saline (0.01 M, 1 × PBS, pH 7.4) was supplied by RICCA Chemical Company, USA. Human urine was purchased from Innovative Research, Inc. USA. LFIA membrane backing cards, nitrocellulose (NC) membrane, glass fiber conjugate pads, and fiber absorbent mats were provided by Millipore Sigma, USA. All chemicals are used without further purification.

*Preparation of Fe-SASC*

Hemin (160 mg) and Zn(NO_3_)_2_·6H_2_O (3.39 g) were added to 150 mL methyl alcohol and dissolved by stirring for 10 min (Solution A). MeIM (3.94 g) was dissolved in 150 mL methyl alcohol to prepare Solution B and then poured Solution B into Solution A with stirring for 1 min. After that, the obtained mixture was allowed to stand overnight at 60°C. The hemin-doped ZIF-8 precursor was collected after centrifugation at 8000 rpm. Finally, Fe-SASC was obtained from the pyrolytic process at 1100°C under nitrogen and ammonia atmosphere for 30 min, respectively. Then acid washing using 3M HCl at 60°C for 4h was performed.

*Preparation* *Fe-SASC labeled antibody*

The Fe-SASC labeled antibody (Fe-SASC-Ab_2,4-D_) was successfully prepared through an amide binding between Ab_2,4-D_ and carboxy-terminated Fe-SASC by EDC/NHS amidation reaction.^1, 2^ Specifically, 1.0 mL of 1.0 mg/mL Fe-SASC was dispersed in 0.05% Nafion (in Ethanol), and the pH value was adjusted to 6 with 0.02 M K_2_CO_3_. Subsequently, the Fe-SASC solution was activated with NHS (4 mg/mL) and EDC (2 mg/mL) by gently shaking for 30 minutes, then washed by centrifuging with PBS three times to obtain the active Fe-SASC. 10 μL of 2,4-D antibody (1 mg/mL in PBS) with active Fe-SASC was incubated at 37 °C for 1 hour and centrifuged at 13000 RPM for three times to remove the unbonded antibodies. Finally, 1% BSA in PBS was used to passivate the products for 30 minutes and the final product was dispersed in 1 mL of PBS and stored at 4 °C for further use.

*Preparation of Fe-SASC labeled competitive LFIA*

The Fe-SASC-LFIA was comprised of the following components: a sample pad, a conjugate pad, a nitrocellulose membrane, an absorbent pad and a backing pad. The sample pad (glass fiber, 17 mm × 30 cm) was treated with a buffer (PBS containing 2% BAS and 2% Sucrose), then dried at 37 °C for 2 h. Test line (1 μL/cm, 30 μL 2,4-D BSA) and control line (1 μL/cm, 30 μL Goat anti-mouse IgG) prepared at different locations on the nitrocellulose membrane (25 mm× 30 cm) by BioJet BJQ 3000 dispenser (BioDot, Irving, California, USA), then dried at 37 °C overnight and stored at 4 °C. The treated sample pad, nitrocellulose membrane and fiber absorbent pad were laminated on a backing card (60 mm × 30 cm). The overlapping length between each pad was approximate 1-2 mm to ensure the migration of the solution. After all components were assembled on the backing card, this card is cut into strips with a width of 4 mm using a BioDot module CM4000 paper cutter (Irvine, CA, USA). These lateral flow strips were then ready to use for 2,4D tests.

*Peroxidase Activity and Kinetics Assay of Fe-SASC*

The 3,3′,5,5′-tetramethylbenzidine (TMB) were used as substrates for the colorimetric reaction of prepared samples. Moreover, TMB was further used as a typical substrate to study the peroxidase activity of the obtained Fe-SASC. Specifically, different amounts of Fe-SASC were dissolved in 0.2 M pH 3.6 NaAc-HAc buffer, respectively. TMB was dissolved in DMSO to 10 mg mL^-1^ and then 100 mL was added to the above solutions. The obtained mixtures were incubated in dark at 37^o^C for 5 min.

Then H_2_O_2_ solution was added to the above mixture to a final concentration of 1M, in which one sample served as background measurement without adding H_2_O_2_. The reaction-time curves of Fe-SASC was plotted using the absorbance at 652 nm against the reaction time. The catalytic activity units (U) was evaluated by detecting the absorbance at 652 nm of a 10 s interval within 700 s. The catalytic activity expressed in units (U) was calculated through the following equation after eliminating background:

SA$\text{=}\frac{\text{V/(Ԑ×l) ×(∆A/∆t)}}{\text{m}}$

In which SA is the specific activity (U mg^-1^); V is the volume of the reaction solution (μL); ε is the molar absorption coefficient of TMB substrate (39,000 M^-1^ cm^-1^ at 652 nm); l is the optical path length through reaction solution (cm); ΔA/Δt is the initial rate (within 1 min) of the absorbance change (min^-1^) and m is the amount (mg) of Fe-SASC in each assay.

The steady-state kinetic measurements of three peroxidase-like single-atomic site catalyst were measured according to the following steps. First, 50 μL of 1 μg mL^-1^ samples in NaAc-HAc buffer (pH 3.6) solution was added to each well, various volumes of TMB solution (10 mg mL^-1^ in DMSO), then certain volumes of H_2_O_2_ solution were added to the reaction mixture to a concertation of 1M. The absorbance at 652 nm was immediately recorded at a 10 s interval within 60 s. The initial rates of the chromogenic reaction to different TMB concentrations were obtained and were fitted with Michaelis-Menten model. Furthermore, Michaelis constant K*_m_* and K*_cat_* were calculated according the following Michaelis-Menten equation:

$$\text{v = }\frac{\text{v}_{\text{max}}\text{[S]}}{\text{K}_{\text{m}}\text{+ [S]}}\text{ }\text{K}_{\text{cat}}\text{=}\frac{\text{υ}_{\text{max}}}{\text{[E]}}$$

where v is the initial rate of the chromogenic reaction, [S] is the TMB concentration and [E] is the concentration of catalysts (M).

To evaluate the peroxidase-like catalytic activity of Fe-SASC against various pH, Fe-SASC was first incubated in buffers with different pH at higher concentration for 2h, then diluted to 10ug/mL using pH 3.6 buffer, and then tested under standard situation. For studying the impact of temperature on peroxidase-like catalytic activity of Fe-SASC, the Fe-SASC was dissolved in pH 3.6 buffer to a concentration of 10 ug/mL, then stored at different temperature for 2h, then tested under standard conditions.





F_IGURE_  S1 XRD pattern of Fe-SASC.


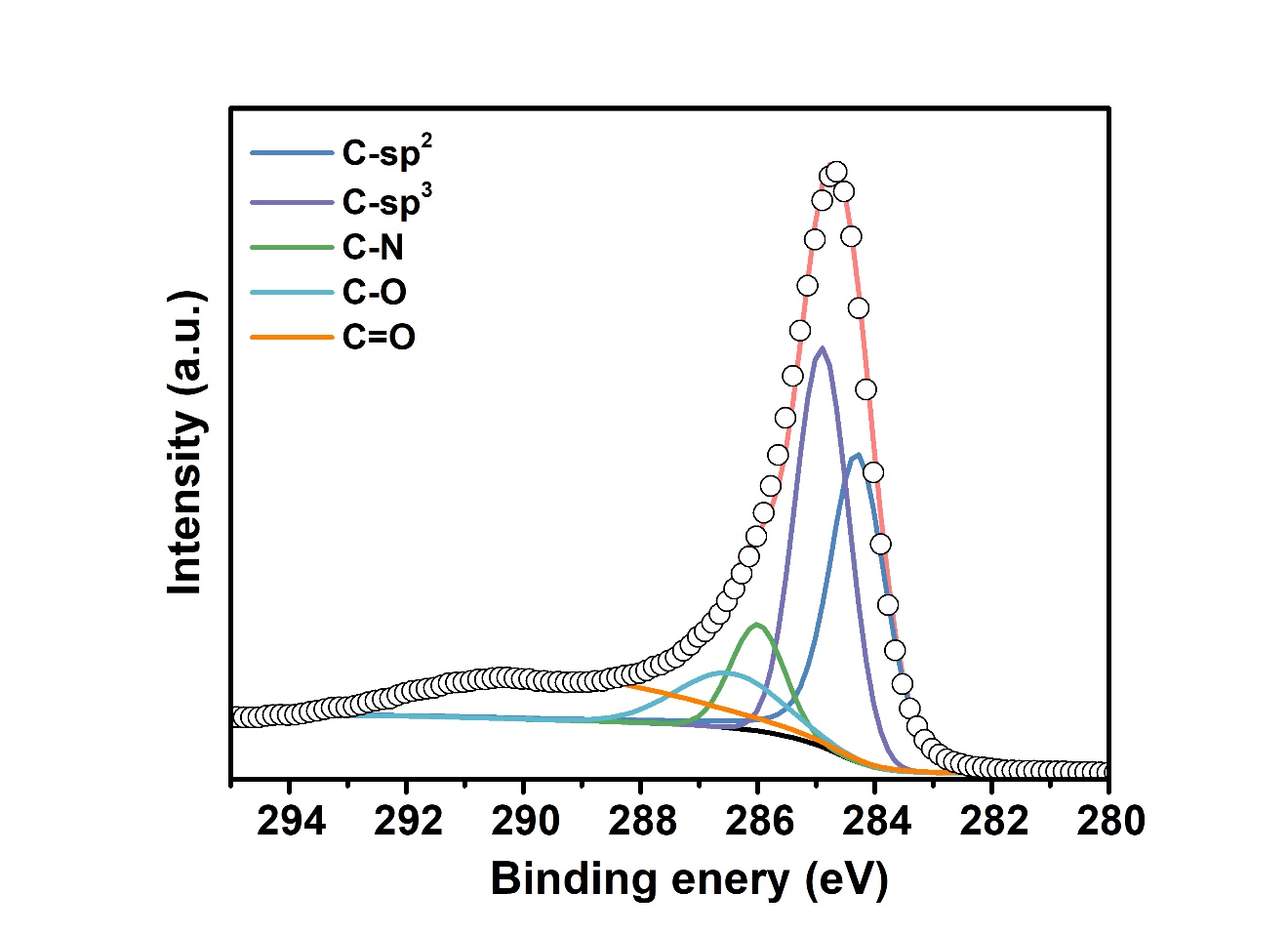


F_IGURE_  S2 High-resolution C 1s spectra of Fe-SASC.





F_IGURE_  S3 The N_2_ adsorption/desorption isotherm curves of Fe-SASC.





F_IGURE_  S4 Pore distribution of Fe-SASC by NLDFT.





F_IGURE_  S5 EXAFS oscillations of Fe-SASC and reference samples of Hemin, FePc, Fe foil, FeO, and Fe_2_O_3_ at k-space.


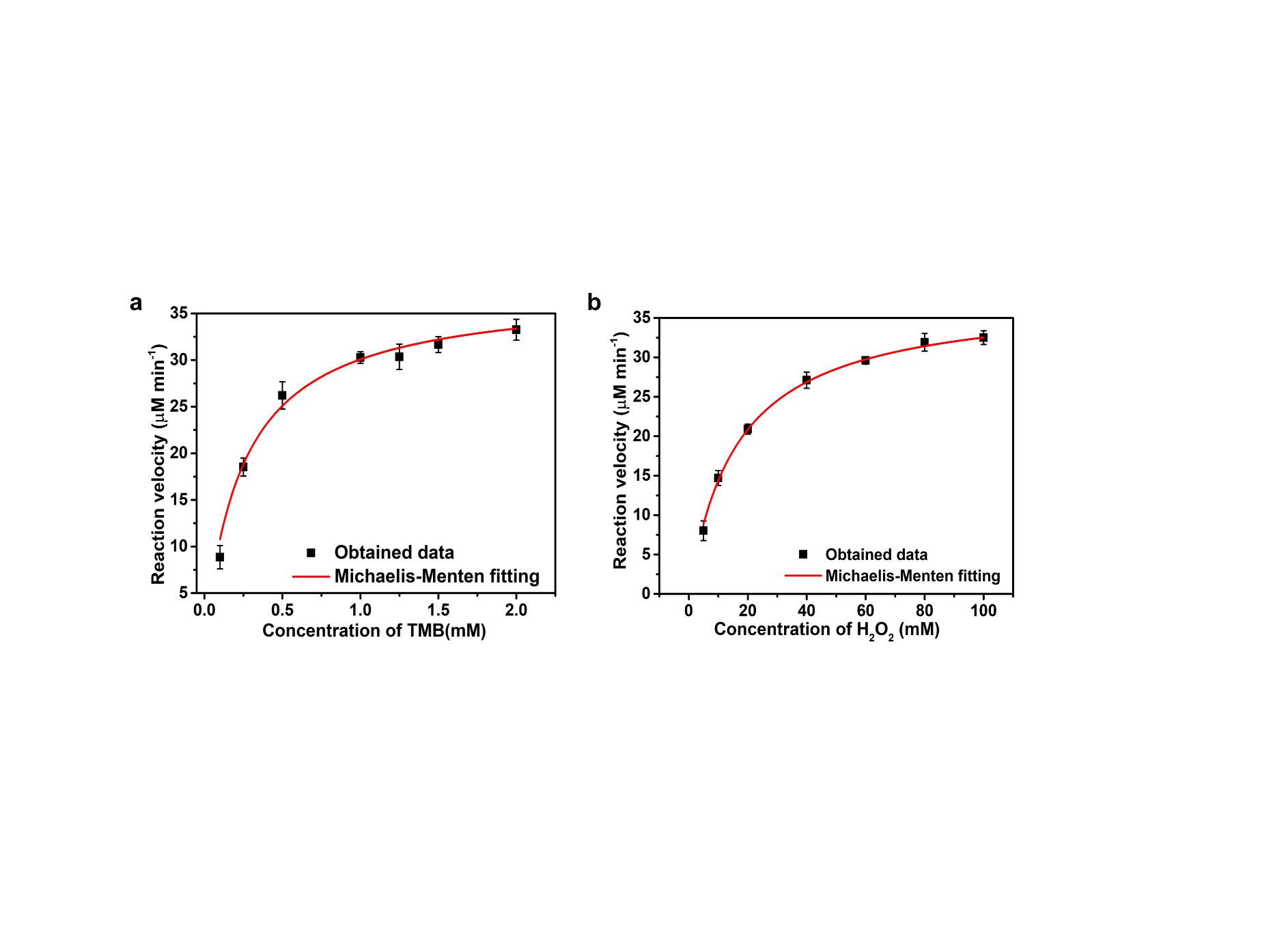


F_IGURE_  S6 Steady-state kinetics curves of Fe-SASC toward (a) TMB and (b) H_2_O_2_.


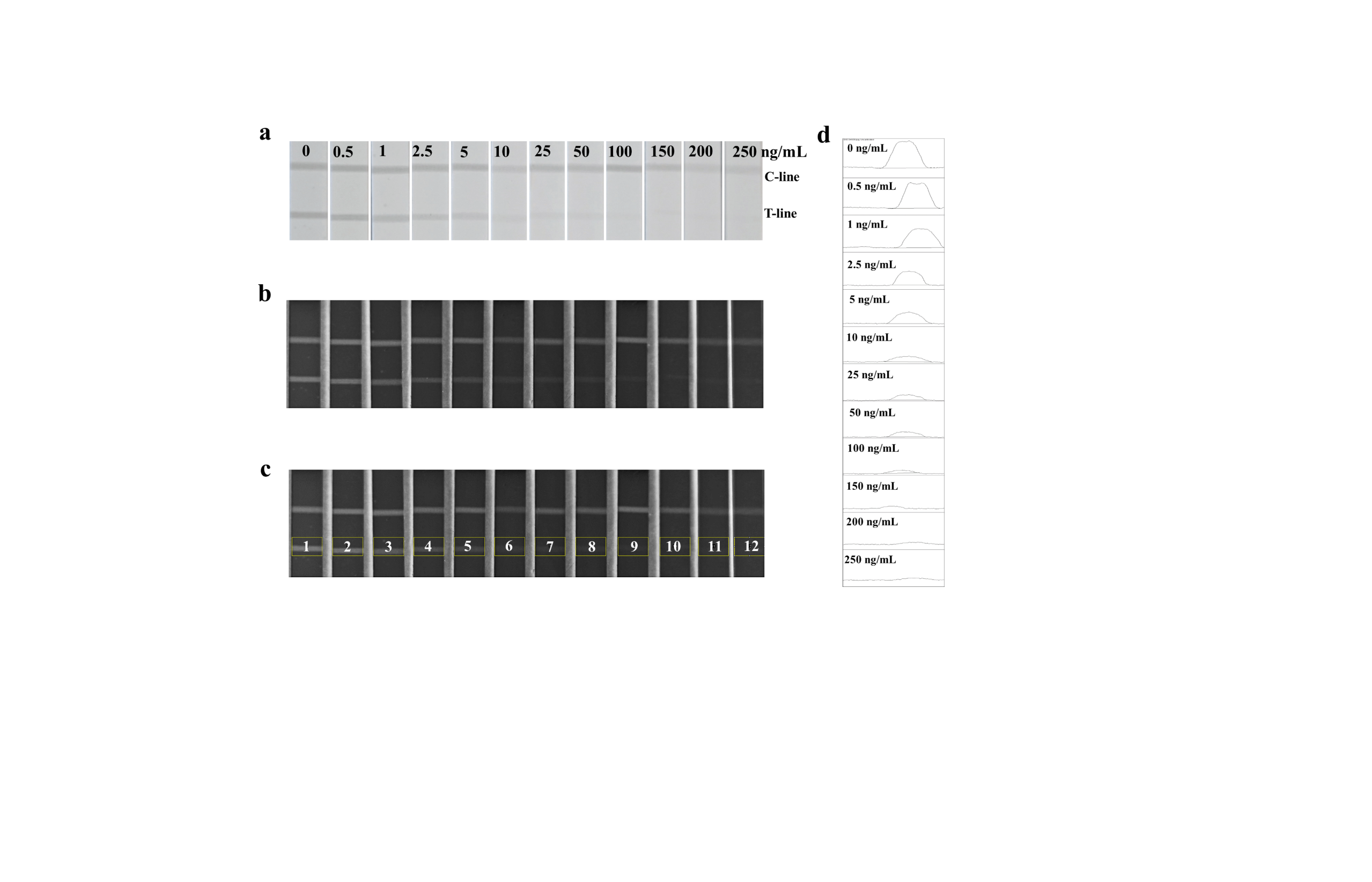


F_IGURE_  S7 Image analysis procedure by Image J software. (a) test strips captured by a camera after 10 min of lateral flow assay, (b) picture converted to an 8-bit grayscale image in Image J software, (c) the Gel Analyzer function of the chosen rectangular regions surrounding the test lines, (d) the signal strength peak generated by the Gel Analyzer function in Image J.


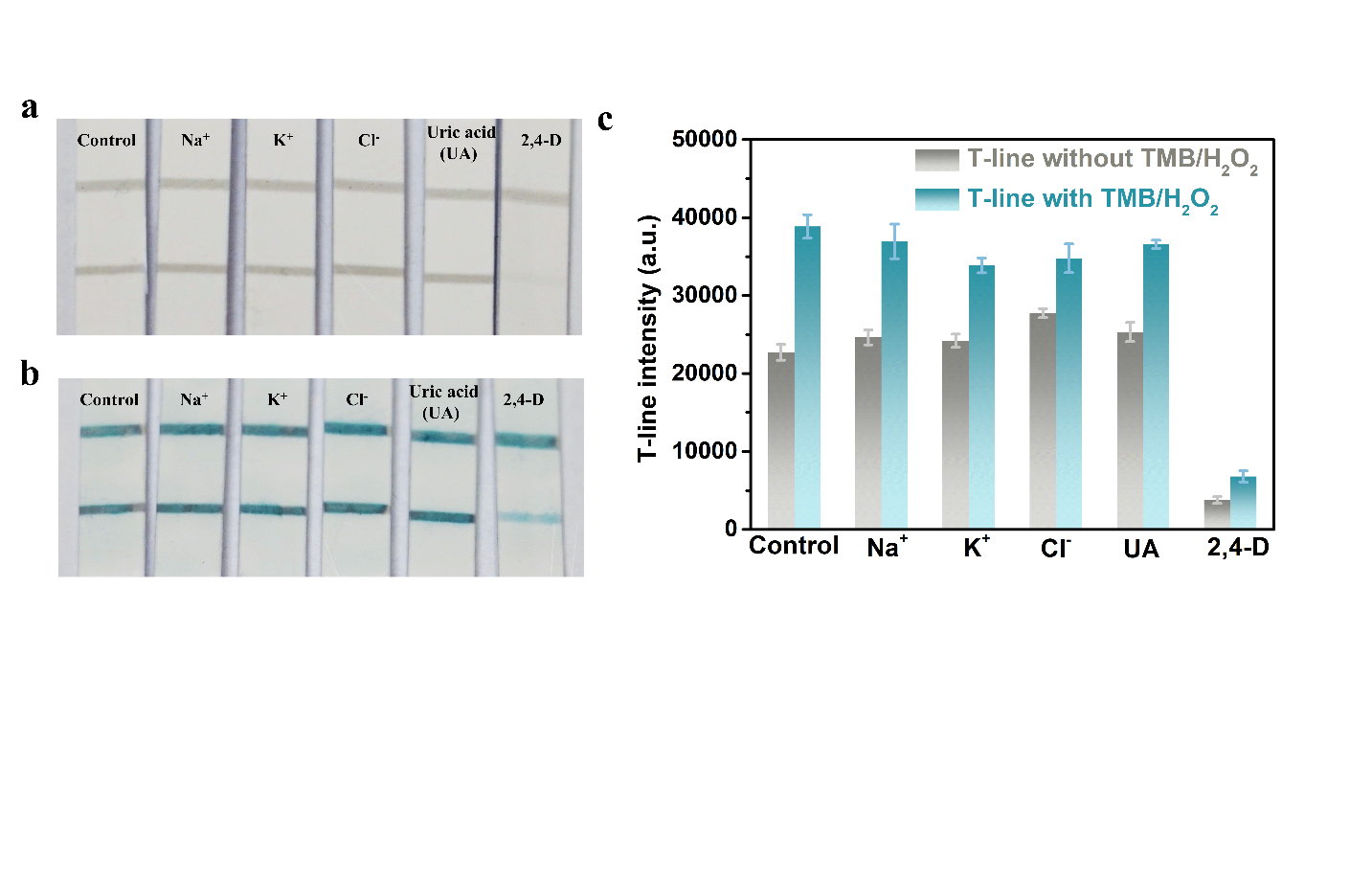


F_IGURE_  S8 (a) and (b) Photographs of Fe-SASC-LFIA towards 2,4-D and interference substances in human urine before and after Fe-SASC enhancement, respectively; (c) Corresponding T-line signal intensities (Concentrations of interfering substances were all 100 ng/ mL, 2,4-D was 25 ng/ mL).

**Table S1** Comparison of peroxidase-mimic activity of Fe-SASC and reported nanomaterials and HRP.

| **Samples** | **Peroxidase-like**  **specific activity (U/mg)** | **Reference** |
| --- | --- | --- |
| Fe-SASC | 46.9 | This work |
| FeNC | 4.09 | [1] |
| FeBNC | 15.41 | [1] |
| SNC nanozymes | 17.5 | [2] |
| Fe_3_C@C/Fe–N–C | 23.01 | [2] |
| NO_2_-MIL-101 | 35.70 | [3] |
| Np-CMNs | 16.2 | [4] |
| Carbon NPs | 3.302 | [5] |
| Au NPs | 1.633 | [5] |
| Natural HRP | 297 | [6] |

**Table S2**. Comparison of steady-state kinetics parameters of Fe-SASC and natural HRP.

| **Materials** | **Substrate** | ***K*_m_**  **(mM)** | ***v*_max_**  **(μM min^-1^)** |
| --- | --- | --- | --- |
| Fe-SASC | H_2_O_2_ | 16.28 | 36.7 |
|  | TMB | 0.24 | 37.81 |
| Natural HRP [7] | H_2_O_2_ | 18.64 | 48.6 |
|  | TMB | 0.427 | 55.49 |

**Table S3** Comparison of our work and other different methods for 2,4-D detetion.

| Techniques | LOD (ng mL^−1^) | Linear Range (ng mL^−1^) | Reference |
| --- | --- | --- | --- |
| Photoinduced electron transfer (PET) | 30.6 | 30.6-7657.5 | [8] |
| SPR immunosensor | 0.5 | 0.5-1000 | [9] |
| ELISA | 1 | 1-80 | [10] |
| Photoelectrochemical sensor | 2.2 | 110-2874 | [11] |
| Fluoroimmunoassay | 0.25 | 0.25-1 | [12] |
| HPLC | 53 | 160-40000 | [13] |
| HPLC | 100 | 100-400000 | [14] |
| Immunoassay | 15 | 50-1000000 | [15] |
| Fe-SASC-LFIA | 0.82 | 1-250 | This work |

**Table S4**. Spiked-recovery test of plasma samples using the proposed Fe-SASC-LFIA in human urine (n=3).

| **Without TMB/ H_2_O_2_** | | | | | | | |
| --- | --- | --- | --- | --- | --- | --- | --- |
| **Sample No.** | **Added (ng mL^-1^)** | | | **Found (ng mL^-1^)** | **Recovery (%)** | | **RSD** |
| 1 | | 5 | 4.72 | | 94.4 | | 5.43 |
| 2 | | 10 | 10.6 | | | 106 | 8.62 |
| 3 | | 20 | 21.9 | | | 109.5 | 7.54 |
| 4 | | 40 | 38.6 | | | 96.5 | 6.36 |

| **With TMB/ H_2_O_2_** | | | | |
| --- | --- | --- | --- | --- |
| **Sample No.** | **Added (ng mL^-1^)** | **Found (ng mL^-1^)** | **Recovery (%)** | **RSD** |
| 1 | 5 | 5.2 | 113.4 | 7.26 |
| 2 | 10 | 9.8 | 98 | 6.92 |
| 3 | 20 | 20.5 | 102.5 | 3.43 |
| 4 | 40 | 39.5 | 98.5 | 5.63 |

**Reference**

1. L. Jiao, W. Xu, Y. Zhang et al., "Boron-doped Fe-NC single-atom nanozymes specifically boost peroxidase-like activity," *Nano Today*, vol. 35, pp.100971, 2020.

<https://doi.org/10.1016/j.nantod.2020.100971>

1. Y. Chen, L. Jiao, H. Yan et al., " Hierarchically porous S/N codoped carbon nanozymes with enhanced peroxidase-like activity for total antioxidant capacity biosensing," *Analytical Chemistry*, vol. 92, pp. 13518-13524, 2020.

<https://doi.org/10.1021/acs.analchem.0c02982>

1. W. Xu, Y. Kang, L. Jiao et al., "Tuning atomically dispersed fe sites in metal-organic frameworks boosts peroxidase-like activity for sensitive biosensing," *Nano-micro Letters*, vol. 12, pp.1-12, 2020. <https://doi.org/10.1007/s40820-020-00520-3>
2. H. Yan, L. Wang, Y. Chen et al., "Fine-tuning pyridinic nitrogen in nitrogen-doped porous carbon nanostructures for boosted peroxidase-like activity and sensitive biosensing," *Research*, vol.2020, 2020. <https://doi.org/10.34133/2020/8202584>
3. B. Jiang, D. Duan, L. Gao et al., "Standardized assays for determining the catalytic activity and kinetics of peroxidase-like nanozymes," *Nature Protocols*, vol. 13, pp.1506-1520, 2018. <https://doi.org/10.1038/s41596-018-0001-1>
4. X. Niu, Q. Shi, W. Zhu et al., "Unprecedented peroxidase-mimicking activity of single-atom nanozyme with atomically dispersed Fe-N_x_ moieties hosted by MOF derived porous carbon," *Biosensors and Bioelectronics*, vol. 142, pp. 111495, 2019. <https://doi.org/10.1016/j.bios.2019.111495>
5. Z. Lyu, S. Ding, N. Zhang et al., "Single-atom nanozymes linked immunosorbent assay for sensitive detection of Aβ 1-40: A biomarker of Alzheimer’s disease," *Research,* vol. 2020, 2020. <https://doi.org/10.34133/2020/4724505>
6. X. Wang, J. Yu, X. Wu et al., "A molecular imprinting-based turn-on ratiometric fluorescence sensor for highly selective and sensitive detection of 2, 4-dichlorophenoxyacetic acid (2, 4-D)," *Biosensors and Bioelectronics*, vol.81, pp. 438-444, 2016. <https://doi.org/10.1016/j.bios.2016.03.031>
7. K. V. Gobi, H. Tanaka, Y. Shoyama et al., "Highly sensitive regenerable immunosensor for label-free detection of 2, 4-dichlorophenoxyacetic acid at ppb levels by using surface plasmon resonance imaging," *Sensors and Actuators B: Chemical*, vol. 111, pp. 562-571, 2005. <https://doi.org/10.1016/j.snb.2005.03.118>
8. Y. Wang, M. M. Zeinhom, M. Yang et al., "A 3d-printed, portable, optical-sensing platform for smartphones capable of detecting the herbicide 2, 4-dichlorophenoxyacetic acid," *Analytical Chemistry*, vol. 89, pp. 9339-9346, 2017. <https://doi.org/10.1021/acs.analchem.7b02139>
9. H. Shi, G. Zhao, M. Liu et al., "A novel photoelectrochemical sensor based on molecularly imprinted polymer modified TiO_2_ nanotubes and its highly selective detection of 2, 4-dichlorophenoxyacetic acid, "*Electrochemistry Communications*, vol. 13, pp. 1404-1407, 201). <https://doi.org/10.1016/j.elecom.2011.08.022>
10. A. Vinayaka, S. Basheer, M. Thakur. "Bioconjugation of cdte quantum dot for the detection of 2, 4-dichlorophenoxyacetic acid by competitive fluoroimmunoassay based biosensor," *Biosensors and Bioelectronics*, vol. 24, pp. 1615-1620, 2009. <https://doi.org/10.1016/j.bios.2008.08.042>
11. S. A. Haeri, S. Abbasi. "Biocoacervation extraction combined with dispersive solid phase extraction using a reversed-phase core-shell magnetic molecularly imprinted sorbent for 2, 4-dichlorophenoxyacetic acid prior to its determination by HPLC," *Journal of the Iranian Chemical Society*, vol. 13, pp. 1993-1999, 2016. <https://doi.org/10.1007/s13738-016-0916-9>
12. X. Chen, H. Zhang, Y. Wan et al., "Determination of 2, 4-dichlorophenoxyacetic acid (2, 4-D) in rat serum for pharmacokinetic studies with a simple hplc method," *PLOS One*, vol. 13, pp. e0191149, 2018. <https://doi.org/10.1371/journal.pone.0191149>
13. X. L. Hu, X. M. Wu, X. Fang et al., "Wang. Switchable fluorescence of gold nanoclusters for probing the activity of alkaline phosphatase and its application in immunoassay," *Biosensors and Bioelectronics*, vol. 77, pp. 666-672, 2016. <https://doi.org/10.1016/j.bios.2015.10.046>
